# Automatic Indoor Reconstruction from Point Clouds in Multi-room Environments with Curved Walls

**DOI:** 10.3390/s19173798

**Published:** 2019-09-02

**Authors:** Fan Yang, Gang Zhou, Fei Su, Xinkai Zuo, Lei Tang, Yifan Liang, Haihong Zhu, Lin Li

**Affiliations:** 1 School of Resource and Environmental Sciences (SRES), Wuhan University, 129 Luoyu Road, Wuhan 430079, China; 2 Institute of Smart Perception and Intelligent Computing, SRES, Wuhan University, 129 Luoyu Road, Wuhan 430079, China

**Keywords:** 3D indoor modeling, curved wall, line regularization, graph cut, point cloud

## Abstract

Recent developments in laser scanning systems have inspired substantial interest in indoor modeling. Semantically rich indoor models are required in many fields. Despite the rapid development of 3D indoor reconstruction methods for building interiors from point clouds, the indoor reconstruction of multi-room environments with curved walls is still not resolved. This study proposed a novel straight and curved line tracking method followed by a straight line test. Robust parameters are used, and a novel straight line regularization method is achieved using constrained least squares. The method constructs a cell complex with both straight lines and curved lines, and the indoor reconstruction is transformed into a labeling problem that is solved based on a novel Markov Random Field formulation. The optimal labeling is found by minimizing an energy function by applying a minimum graph cut approach. Detailed experiments were conducted, and the results indicate that the proposed method is well suited for 3D indoor modeling in multi-room indoor environments with curved walls.

## 1. Introduction

Recently, 3D building modeling has been increasingly requested for a variety of applications, such as building information models (BIM) [1], indoor navigation and positioning, emergency services, smart building, and architectural planning and simulations. The modeling of as-built constructions can range from mesh and computer-aided design (CAD) models to object-based parametric models. Mesh representations are focused on creating visually realistic models. CAD models focus on representing geometry digitally for the purpose of design and simulation. In contrast, semantic information, as well as parametric information with a defined data structure, is added to the geometry of object-based parametric models. The Open Geospatial Consortium (OGC) CityGML [2] and industry foundation classes (IFC) [3] are commonly used for semantically rich representations of buildings, in which, apart from detailed geometry, semantics and topology play an important role [4].

Advances in laser scanning systems have provided an efficient way to collect 3D point clouds of both indoor and outdoor scenes. However, this procedure is challenging due to complex building layouts and the high presence of objects such as furniture and wall hangings that cause clutter and occlusions [4]. Furthermore, the procedure is challenging due to noise. Recent works have exploited prior knowledge about building structures to achieve robustness and transform scene reconstruction into an indoor space decomposition problem. Despite the rapid development of 3D reconstruction methods for building interiors from point clouds, the reconstruction of curved walls in multi-room environments is still not resolved.

Many works have utilized the Manhattan world (MW) assumption [4], which holds true for many indoor scenes that contain man-made architectural structures. The MW assumption assumes that most man-made structures can be approximated by planar surfaces that are parallel to one of the three principal planes of a common orthogonal coordinate system. Obviously, the MW assumption is not suitable for indoor spaces with curved walls. One direct method for addressing curved walls is to use piecewise straight lines to approximate a curved line [5,6], but this method is not always appropriate for the following two reasons. On the one hand, the use of a few piecewise straight lines to approximate a curved line will lead to an unsmooth boundary of rooms [5]. On the other hand, the use of too many piecewise straight lines will lead too many small cells in the cell complex. It is difficult to arrange a label to such small cells. Furthermore, it is not trivial to detect curved surface walls in multi-room indoor environments [7,8]. Although slices are often used to simplify detection work, detected curved lines are often scattered and broken into pieces and vary in length, which is not suitable for modeling problems. It is also difficult to detect straight and curved lines separately, especially in the case of noisy point clouds.

In this work, a novel straight and curved line tracking method with a straight line test was proposed to detect straight and curved walls. Robust parameters are used, and a novel straight line regularization method is achieved by using constrained least squares. Room space decomposition with curved walls is cast as an energy minimization problem that partitions the indoor space into separate semantic entities. A novel Markov Random Field (MRF) formulation is proposed, and the energy function is efficiently minimized using an existing graph cut method. The proposed method is performed without a priori knowledge of viewpoints, and it is not restricted to the strong MW assumption. The assumption of this study is that floor and ceilings are horizontal and walls are orthogonal to the floor.

The remainder of this paper is organized as follows. Important related works are introduced in Section 2. The semantic definition of indoor space, structural constraints and the proposed method are described in Section 3. Experiments and the discussion are presented in Section 4. Finally, conclusions are drawn in Section 5.

## 2. Related Works

The indoor reconstruction method can be classified as a structural element extraction method and a space decomposition-based method. The former research attempts to classify the point cloud of indoor scenes and extract structural elements. A method for the planar 3D modeling of building interiors from point clouds was proposed in [9]. This method divides indoor points into ceilings, floors, walls, and other small architectural structures according to the normally computed principal component analysis (PCA) algorithm, and random sample consensus (RANSAC) is employed to fit the detected planar primitives. Furthermore, predominantly planar surfaces, such as walls, floors, and ceilings, were modeled [10], despite the presence of significant clutter and occlusion, which occur frequently in natural indoor environments. Shi et al. [11] proposed a semantic geometric modeling method to reconstruct 3D building models with semantic information from the unstructured 3D point cloud. The method enables the accurate detection of wall surfaces.

The structural element extraction methods for building structure elements cannot construct complete wall surfaces due to missing data and the existence of occlusion in scanned point clouds. Thus, the space decomposition-based method exploits prior knowledge about building structures to achieve robustness and transform the scene reconstruction into an indoor space decomposition problem.

By initializing the domain of the point clouds in the XOY plane, the k-medoids algorithm was applied to cluster subsampled pixels [12], and room segmentation was then formulated as a clustering problem. Previtali et al. [4] proposed an automatic method for modeling MW indoors from occluded point clouds using graph cut and ray tracing. However, these two methods use a strict MW assumption, which limits the adaptability of the method.

In the work by Oesau et al. [13], the point cloud is partitioned by taking horizontal cross sections from which lines are fitted. A 2D cell decomposition is performed and is then stacked to partition the 3D space. The surfaces are extracted from the 3D cell decomposition by labeling the cells as either empty or solid space, and a set of faces between adjacent labeled cells is constructed for the final model as a surface mesh.

Mura et al. [14] proposed an automatic method for room detection and reconstruction that starts by projecting the entire point cloud onto the XOY plane. The projected candidate walls are represented by a small number of lines. Then, a cell complex is built from the intersections of the representative lines, the edges of which are weighted according to the likelihood that the walls are real. Finally, diffusion distances, which are computed on the cell graph of the complex, are used to derive the iterative cell clustering, which enables the extraction of separate rooms. This method can be used in cluttered indoor environments with complex room layouts.

A decomposition and reconstruction strategy [15] was then employed to create indoor structural elements on the floor plan. This method can address the reconstruction of both inner and outer walls by solving the optimization energy problem using graph cut. It utilizes a hierarchical clustering method to obtain initial labels and a merging method to refine over-segmented rooms.

The reconstruction task [6] is formulated as a multiclass labeling problem using energy minimization. A greedy fashion is used to sample viewpoints, and simulated viewpoints are obtained automatically. The room segmentation algorithm can lead to over-segmentation in cases where imaginary walls are inferred, and such regions need to be merged in a post-processing step.

As the above studies are based on either the constrained MW assumption or the detection of only planar walls, none has proven to be an efficient method for indoor reconstruction with curved walls. The author in [6] proposed an idea to find piecewise linear approximations of the projected curve and insert each individual line segment into the cell complex to deal with curved wall surfaces. However, no experiment was conducted in their research. In theory analysis, the idea can work in some cases (very few curved walls) but with some limitations. The approach relies on the proper length of each piecewise linear approximation to represent the curved wall lines. However, straight line detection methods do not work well for curved line detection. Furthermore, the author did not give any curved line detection method. The use of just a few piecewise straight lines will lead to unsmooth room boundaries. However, the use of too many piecewise straight lines will lead too many small cells in the cell complex.

To the best of our knowledge, no previous research has attempted to solve the problem of indoor reconstruction from point clouds in multi-room indoor environments with curved walls. It is not trivial to detect curved surface walls in multi-room indoor environments. To simplify the work of curved surface wall reconstruction, 2D slices are often used [7,16]. Because this work restricts the assumption that walls are orthogonal to the ground, the slice method is proper. Curved wall surface detection becomes a 2D curved line detection problem. Line feature detection is widely used in 2D images [17,18]. The Canny edge detector is often used for straight line feature detection. The image-based method for curved line detection often contains three steps, e.g., transformation to a binary image, image thinning and line detection. Some methods, such as the RANSAC [19,20], Hough transforms method [21] and least-square (LS) methods [22], directly detect straight lines in point clouds. LS methods are often used for line fitting or regular shape (e.g., circle, ellipse) fitting. Structural lines in the indoor environment contain not only straight lines but also curved lines. Yang et al. proposed a curved line detection method [23] that first extracts edge points and then employs a tracing step. Contour curves have been computed for rapid prototyping model generation via adaptive slicing, data point reduction and B-spline curve fitting [7]. However, the method only works for closed curves. Some researchers have achieved the detection of exterior building boundaries, but this method is not proper for multi-room indoor environments because exterior building boundaries are often closed, while edge lines are discrete in indoor environments. These methods often use the tracing method for curved line detection. As noise and occlusions exist, the scan point clouds on the wall are often incomplete. The detected curved lines are often scattered and vary in length. The lines are often broken. Such detected curved lines are not suitable for modeling problems. Although slices are often used to simplify the work, it is still not easy to detect straight and curved lines separately, especially in the case of noise point clouds.

## 3. Proposed Method

### 3.1. Overview

In this section, we introduce our automatic indoor 3D reconstruction framework. First, we define straight lines and curved lines. A straight line is a succession of points that are aligned in the same direction. In other words, when going from one point to another, the direction never changes. In contrast, the points of a curved line change direction from one point to the next [24]. The input into this work is a 3D point cloud; the $p_{i}\in P,{\;p}_{i}=\left\{x_{i},y_{i},z_{i}\right\}$ coordinates of each point are given via the world coordinate system.

**Figure 1:**
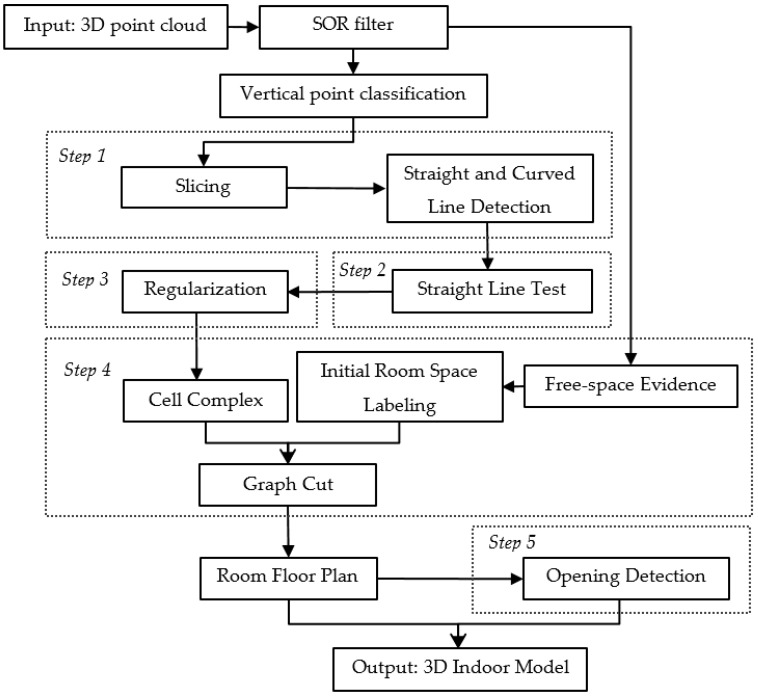
Working flowchart of the proposed method.

We now describe the components that compose our method. A visual overview of the method is given in Figure 1 and consists of five main steps: (1) straight line segment and curved line detection, (2) straight line test, (3) regularization, (4) room space decomposition and (5) opening detection.

### 3.2. Straight and Curved Line Detection

The proposed straight and curved line detection method is described in detail in this section. The preprocessing step uses the statistical outlier removal (SOR) method [25] to clean outliers. This step rejects the points that are farther than the average distance plus a number of times the standard deviation. The vertical attribute $a_{h}=\left|n_{i}\cdot n_{z}\right|$ is used to measure the deviation of the unit normal $n_{i}$ to point $p_{i}$ with respect to the vertical axis, where $n_{z}$ denotes a unit vector along the z coordinate axis [26]. If $a_{h}<\varepsilon$, the point is classified as vertical point. The variable ϵ, which is the cosine value of the angle threshold, is the threshold used to distinguish a vertical point. If the angle threshold is set to 90°±1°, ϵ=cos(90°±1°). Thus, flat structural elements, such as tables and beds, are rejected.

**Figure 2:**
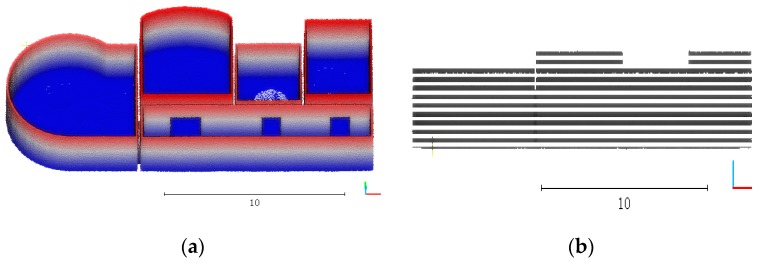
A point cloud split into a set of horizontal slices. (**a**) original point cloud; (**b**) sliced point cloud.

**Figure 3:**
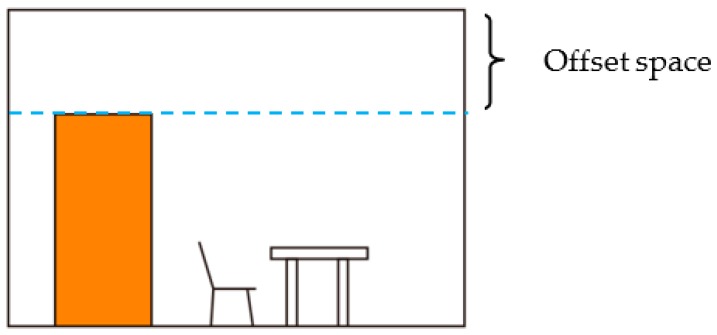
Objects on the floor. The offset space is determined by door height experientially.

The vertical point cloud is then split into a set of horizontal slices, as shown in Figure 2. Generally, the indoor objects like furniture often located on the floor. The indoor space near the ceiling is often free space. Thus, we follow the concept of offset space proposed by [27]. A door will be lower than the height of the wall in which it is contained. As shown in Figure 3, the slices located in the offset space are selected in priority. The offset space can be determined by door height experientially.

**Figure 4:**
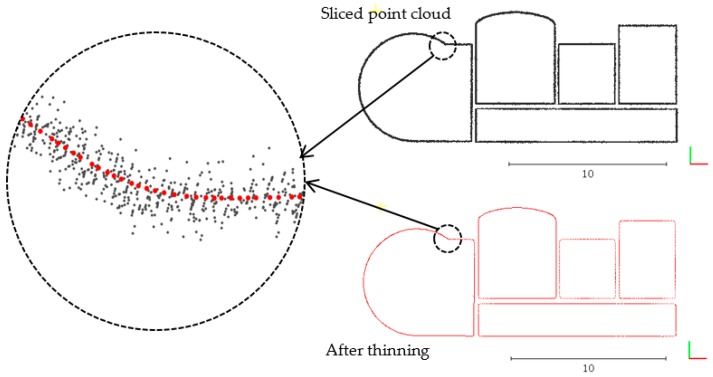
The sliced point cloud is thinned by the mean-shift algorithm.

The straight and curved line detection method contains two steps. Because noise exists, the sliced wall should be thinned first. Mean-shift is a mode-seeking algorithm that searches for the maximum densities of local neighborhoods from discrete points [28]. The algorithm is iterated to obtain a stable result, that is, a certain number of iterations are reached or all points move to the location of the local maximum density in the support neighborhood. To obtain a stable result, the slice thinning algorithm runs k times, with the search radius increasing 1.5 times; and the search radius should be smaller than the thickness of a wall. The employed version of the regularized mean-shift formula [28] is:

(1) $$x_{i}^{k+1}=\frac{\sum_{j\in J}p_{j}^{k}\alpha_{\mathrm{ij}}^{k}}{\sum_{j\in J}\alpha_{\mathrm{ij}}^{k}}+\lambda \frac{\sum_{i’\in I\backslash \left\{i\right\}}\left(x_{i}^{k}-x_{i’}^{k}\right)\beta_{\mathrm{ii}’}^{k}}{\sum_{i’\in I\backslash \left\{i\right\}}\beta_{\mathrm{ii}’}^{k}}$$

(2) $$\alpha_{\mathrm{ij}}=\frac{\theta \left(||x_{i}-p_{j}||\right)}{\left||x_{i}-p_{j}|\right|},{\;\beta }_{\mathrm{ii}’}=\frac{\theta \left(||x_{i}-x_{i\prime }||\right)}{||x_{i}-x_{i\prime }||{}^{2}}$$

(3) $$\theta \left(\left||x_{i}-p_{j}|\right|\right)=e^{-||x_{i}-p_{j}||{}^{2}/R^{2}},\;\lambda =\mu \sigma \left(x_{i}^{k}\right)$$

where the first item is the classical mean-shift algorithm; the second item is a regularized term to prevent further accumulation once points are already contracted onto their local center positions; λ is a balancing constant between the two items; and μ=0.35 is chosen empirically [29]. Using the above formula, the mean-shift algorithm clusters the sampling points based on the resampling of the original point. In this study, 10% of the input points are selected as initial samples. The sliced point cloud thinned by the mean-shift algorithm is shown in Figure 4.

**Figure 5:**
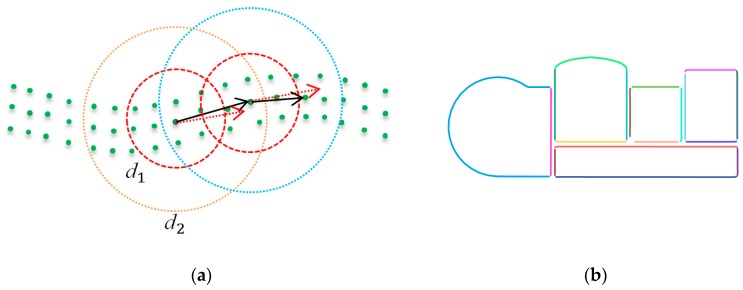
(**a**) Illustration of straight and curved line tracing; (**b**) wall line detection results.

The wall line tracing algorithm contains the following steps:

Step 1: Build the KD tree for the sliced point cloud.
Step 2: Randomly select a point from the point cloud. Search the nearest neighbor within a radius less than the threshold $d_{2}$ (Figure 5). The eigenvalues λ1≤λ2≤λ3 and the corresponding eigenvectors ${\vec{e}}_{1},{\vec{e}}_{2},{\vec{e}}_{3}$ of the covariance matrix Σ are computed for each selected point. The eigenvector ${\vec{e}}_{3}$ is selected as the search direction. The eigenvector −${\vec{e}}_{3}$ is selected as the reverse search direction.
Step 3: The candidate points within a radius less than the threshold $d_{1}$ (Figure 5) are marked as visited. Suppose that the candidate point in the sphere has a minimum angle with the principal direction of the seed point. The minimum angle is less than Δθ (40° in this study). The point is selected as the new seed point. The seed point is marked as a vertex point.
Step 4: If the entire vertexes are traced, the two opposed lines are merged.
Step 5: Go to Step 2 until all the points in the sliced point cloud are marked as visited and vertex points.

### 3.3. Straight Line Test

The traced wall lines consist of a set of vertexes. The distance between adjacent vertexes is relatively even. After the wall line tracing step, the detected wall lines are classified as straight lines and curved lines. In this step, only x, y coordinates of each vertex is used in the algorithm, so the description below is 2-dimensional. A straight line can be defined as a pair $\left(p,\vec{v}\right)$, where p is a point on the line, and $\vec{v}$ is the line’s direction vector. p is estimated as the median of the positions of all vertexes in the polyline. $\vec{v}$ is a normalized direction vector whose value is determined by the median of each component line segment of the polyline.

(4) $$p=\;{\left[\mathrm{median}\left(P_{x}\right),\;\mathrm{median}\left(P_{y}\right)\right]}^{T}$$

(5) $$\vec{v}={\left[\mathrm{median}\left(d_{x}\right),\mathrm{median}\left(d_{y}\right)\right]}^{T}$$

**Figure 6:**
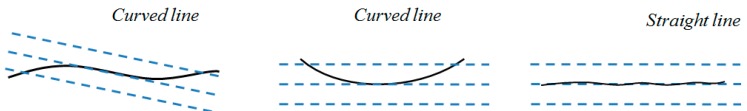
Examples of straight line test results.

The distance of each vertex to the estimated straight line is obtained using Equation (6); we compute the angle $\theta_{i}$ between the vector from each point $P_{i}$ to the center point p and the straight line direction vector using Equation (7). If Condition 1 and Condition 2 are satisfied, the line is a straight line; otherwise, it is a curved line. Three examples are shown in Figure 6.

(6) $$d_{i}=\left|\left(P_{i}-p\right)\cdot \vec{v}\right|$$

(7) $$\cos\left(\theta_{i}\right)=\frac{\left(P_{i}-p\right)\cdot \vec{v}}{\left||P_{i}-p||\cdot ||\vec{v}|\right|}$$

(8) $$\mathrm{Condition}\;1:\;\cos\left(\theta_{i}\right)>\cos\left(\Delta \theta ’\right)||\cos\left(\theta_{i}\right)<\cos\left(\pi -\Delta \theta ’\right)\;\mathrm{Condition}\;2:{\;d}_{i}<\Delta d\;\mathrm{if}\;\mathrm{Condition}\;1\;\&\;\mathrm{Condition}\;2,{\;L}_{i}\;\mathrm{is}\;a\;\mathrm{straight}\;\mathrm{line};\mathrm{otherwise},{\;L}_{i}\;\mathrm{is}\;a\;\mathrm{curved}\;\mathrm{line}.$$

### 3.4. Regularization

The typical indoor structures in man-made buildings, i.e., walls, ceilings, floors, doors, and windows, are composed of straight lines; most of these structures are parallel or orthogonal. Because noise exists in the point cloud, regularization is necessary.

The above step classifies the wall lines into a set of straight line segments and a set of curved lines. The initial value for each straight line segment parameter is obtained using the median point coordinates and median line direction. The line parameter for each straight line is represented as $\left(p_{i},{\vec{v}}_{i}\right),i=0,1,2,\cdots$. Provided that the first line is the reference $L^{\mathrm{ref}}$ (the longest line segment, or a line including the greatest number of point clouds), the conditional rules for the other orthogonal and parallel lines $L_{i},i=1,2,3,\cdots$ are defined as:

(9) $$\{\begin{array}{c} \mathrm{if}\cos\left(\theta \right)>\cos\left(\Delta \theta \right)||\cos\left(\theta \right)<\cos\left(\pi -\Delta \theta \right),{\;L}_{i}\;\mathrm{is}\;\mathrm{parallel}.\\ \mathrm{if}\;\cos\left(\theta \right)<\sin\left(\Delta \theta \right),L_{i}\;\mathrm{is}\;\mathrm{orthogonal}.\;\\ \cos\left(\theta \right)=\frac{{\vec{v}}^{\mathrm{ref}}\cdot {\vec{v}}_{i}\;}{\left||{\vec{v}}^{\mathrm{ref}}||\cdot ||{\vec{v}}_{i}|\right|} \end{array}$$

For other lines, the angle between the line and reference line is θ. Δθ is the empirically determined threshold (10° was used in this work). If condition 1 is satisfied, the line is supposed to be parallel to the reference line. If condition 2 is satisfied, the line is supposed to be orthogonal to the reference line.

In the regularization process, to obtain the optimum parameter for each straight line, the constrained LS method is used. The wall-surface lines in the indoor environment are always orthogonal or parallel to each other. For line representation, the line segment is represented by Equation (10).

(10) ax+by+c=0

The reference line is represented by:

(11) $$L^{\mathrm{ref}}\left(a_{0},b_{0},c_{0}\right)={\;a}_{0}x+b_{0}y+c_{0}$$

If a straight line is parallel to the reference line, then:

(12) $$L_{i}\left(a_{i},b_{i},c_{i}\right)={\;a}_{i}x+b_{i}y+c_{i}=a_{0}x+b_{0}y+c_{i}$$

If a straight line is orthogonal to the reference line, then:

(13) $$L_{i}\left(a_{i},b_{i},c_{i}\right)={\;a}_{i}x+b_{i}y+c_{i}=b_{0}x-a_{0}y+c_{i}=a_{0}\left(-y\right)+b_{0}x+c_{i}$$

This results in a linear system Ax=b. Let A be the matrix of the linear system, x denote the vector of unknowns and b=∅ for the right-hand side.

$$x={\left(c_{0},c_{1},\cdots ,c_{i},a_{0},b_{0}\right)}^{T}$$

**Figure 7:**
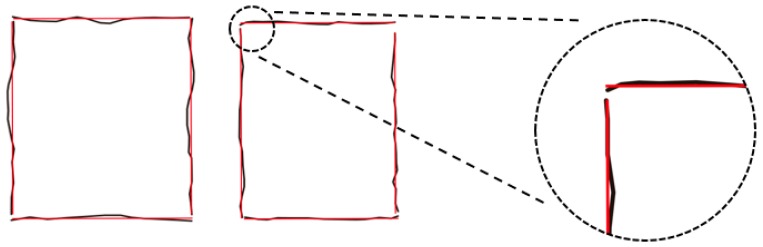
Examples of straight line regularization.

Use QR decomposition of A and reduce this problem to solve a small system. The solution is given by the corresponding singular vector. After the orthogonal and parallel principles are applied, the collinear principle is used to obtain the mean value for collinear lines. The straight line regularization algorithm is presented in Algorithm 1. Examples for straight line regularization can be seen in Figure 7.

**Algorithm1.** Straight Line Regularization Algorithm.Input:L: straight lines $L_{\mathrm{new}}$: adjustment straight linesA: the matrix of the linear systemInitialize: $L_{\mathrm{new}}\leftarrow \emptyset$;//array(1) $L^{\mathrm{ref}}$ = find_longest_line(L);(2) $\mathrm{for}\;\mathrm{each}\;(L_{i}\in L$)(3) $\cos\left(\theta \right)=\frac{{\vec{v}}^{\mathrm{ref}}\cdot {\vec{v}}_{i}\;}{\left||{\vec{v}}^{\mathrm{ref}}||\cdot ||{\vec{v}}_{i}|\right|}$;(4)  if cos(θ)>cos(Δθ)||cos(θ)<cos(π−Δθ)(5)  $L_{i}$ is parallel;(6)  if cos(θ)<sin(Δθ)(7)  $L_{i}$ is orthogonal;(8) end for(9) $\mathrm{for}\;\mathrm{each}\;(L_{i}\in L$)(10)  $\;\mathrm{if}\;L_{i}$ is parallel(11)   Construct A using Equation (12);(12) $\;\mathrm{else}\;\mathrm{if}\;L_{i}$ is orthogonal(13)   Construct A using Equation (13);(14)  End if(15) end for(16) $x=\arg\underset{x}{\;\min}\;\overset{N}{\underset{i=1}{\sum }}{\left|\left|\mathrm{Ax}-b\;\right|\right|}_{2}^{2}\;$.;//solving a linear system(17) $\mathrm{for}\;\mathrm{each}\;(L_{i}\in L$)(18) $\;\mathrm{if}\;L_{i}$ is parallel(19)   $L_{i}^{’}\leftarrow \left(c_{i},a_{0},b_{0}\right),L_{\mathrm{new}}\leftarrow L_{\mathrm{new}}\cup {\;L}_{i}^{’}$;(20)  $\;\mathrm{else}\;\mathrm{if}\;L_{i}$ is orthogonal(21)   $L_{i}^{’}\leftarrow \left(c_{i},b_{0},-a_{0}\right),L_{\mathrm{new}}\leftarrow L_{\mathrm{new}}\cup {\;L}_{i}^{’}$;(22)  end if(23) end for(24) $\mathrm{return}\;L_{new}$;

### 3.5. Room Space Decomposition

In this study, we consider room space reconstruction as a labeling problem. A decomposition and merges strategy is used in this research. First, the 2D space is partitioned by wall straight lines, and the cell complex is constructed by subdividing the plane into zero-dimensional, one-dimensional and two-dimensional cells, called vertices, edges and polygons, respectively. Then, the polygon feature class is further subdivided with curved walls. As the detected curved lines may be shorter than their true length, it is necessary to extend the start and end line segments with a certain length. The labeling problem addresses assigning a label from a set of labels to each of the cells in the cell complex. The optimum labeling is achieved by assigning each cell a label from the label set to minimize an objective function. An undirected graph G=〈v,e〉 is defined as a set of nodes v and a set of undirected edges e to encode the set of cells to the label set L.

In contrast to our previous work [5], in the present study, indoor space decomposition is defined as an energy minimization problem. The energy is minimized on a dual graph by associating data term with the vertices (representing the faces in the cell complex) and smooth terms with the edges. The graph edges are used to model the relations between pairs of adjacent nodes $v_{i}$ and $v_{j}$, thus enabling the modeling of contextual relations. Therefore, each cell $v_{i}$ is linked to its neighbors ($v_{j}\in N$) in 2D by edges, where N is the set of neighborhood links. The energy function for the labeling problem is defined as:

(14) $$U\left(l\right)=\underset{i\in v}{\sum }D_{i}\left(l_{i}\right)+\underset{i,j\in N}{\sum }V_{\mathrm{ij}}\left(l_{i},{\;l}_{j}\right)\cdot T\left(l_{i}\neq l_{j}\right)$$

#### 3.5.1. Data Term

The unary data term describes the likelihood that a cell belongs to a label. The unary potential links data to the class labels and determines the most likely label for a single node. The initial value is obtained from the labeling results on a free-space raster using morphological room segmentation [27,30]. The free-space raster is generated by orthographically projecting the point cloud onto the XOY plane. The wall-occupied evidence is generated by orthographically projecting the selected sliced point cloud onto the XOY plane. The morphological algorithm segments the free-space evidence raster into semantically meaningful regions. The minus operator [31] between the free-space evidence and wall-occupied evidence is also utilized to obtain refined free-space evidence [5].

The assignment of the unary data term is achieved by using the Monte Carlo method, which randomly samples N points within each cell complex; the value of each point is extracted from the labeled room raster. In addition, a center point in each cell is added to the random point set in case there are no points sampled inside a small cell.

(15) $$D_{i\in v}\left(l_{i}\right)=\left(1-\frac{\mathrm{count}\left(\mathrm{pixel}=l_{i}\right)}{N+1}\right)*\mathrm{area}\left(\mathrm{cell}\right)$$

#### 3.5.2. Smoothness Term

The smooth term associated with each edge intuitively describes the likelihood that two cells are contained within the same room. A larger likelihood between two cells indicates a weaker linking between them. When two cells are separated by a wall, the cells are unlikely to be in the same room. Taking $w_{\mathrm{cell}}$ for the cell complex edge and w for the original wall segment, Ambruş et al. [6] obtained a smooth term between two cells separated by edge e, i.e., $1-\left|w_{\mathrm{cell}}\cap w\right|/\left|w_{\mathrm{cell}}\right|$. The overlap between straight segments is easy to calculate, but it is difficult to calculate the overlap between curved lines. Therefore, the wall-occupied evidence raster was used for smooth term calculation. The smooth term is defined as the ratio between pixels occupied by the original wall segment and pixels in the cell complex edge.

(16) $$V_{i,j\in N}\left(l_{i},{\;l}_{j}\right)=1-\frac{\mathrm{count}\left(\mathrm{pixel}=\mathrm{occupied}\;\mathrm{and}\;\mathrm{pixel}\in w_{\mathrm{cell}}\right)}{\mathrm{count}\left(\mathrm{pixel}\in w_{\mathrm{cell}}\right)}$$

**Figure 8:**
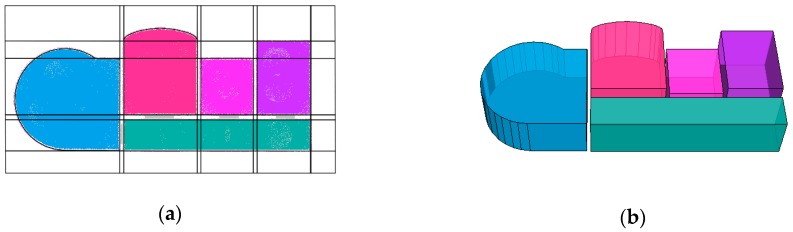
(**a**) Cell complex overlapping with the initial room segmentation raster and (**b**) constructed room spaces.

The comparison predicate $T\left(l_{i}\neq l_{j}\right)$ is 1 if the argument is true and 0 otherwise. An approximate solution can be found by minimizing the objective function by applying a minimum graph cut approach. This energy minimization problem is solved through the α-β swap algorithm [32]. A room floorplan is generated after room space decomposition. Finally, the histogram method is used to obtain the average height from the floor to the ceiling for each room [5]. As shown in Figure 8a, the cell complex is added to the initial room segmentation raster layer. The constructed room spaces are shown in Figure 8b.

### 3.6. Opening Detection

In this step, we introduce a wall opening element detection method that detects doors and windows as holes in the point clouds of wall planes. With a mapping function, both straight walls and curved walls can be mapped to wall point evidence images, which are inverse binary images created by projecting the points on the wall to the wall surface. First, the points on the curved wall are projected to the wall point evidence image. The *x* axis is the distance to the start point of a curved line, and the *y* axis is the height of each point on the wall.

f(d,h)↔f(u,v)

Second, the hollow areas on the wall are extracted using the U-V direction region growing. The algorithm contains two main components. The first component consists of obtaining seed points from the last row (i.e., seed selection in the V-direction). The second component consists of obtaining the un-labeled pixels of the current row and performing growth in the row (i.e., growth in the U-direction). After obtaining all of the clusters, statistical calculations are performed on the rows and columns of each cluster. The median values in the horizontal and vertical directions of each cluster, which can be treated as the width and height of an opening, respectively, are then obtained.

**Figure 9:**
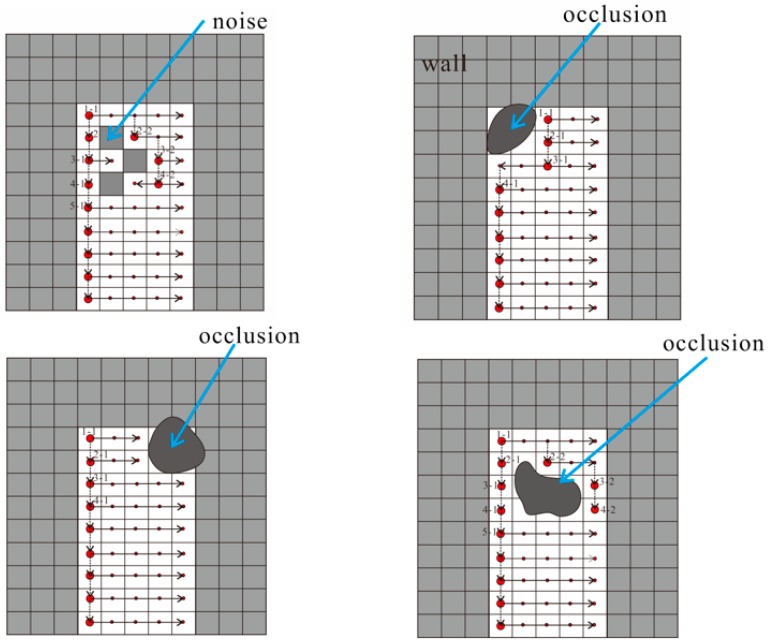
Four examples for U-V direction region growing.

**Figure 10:**
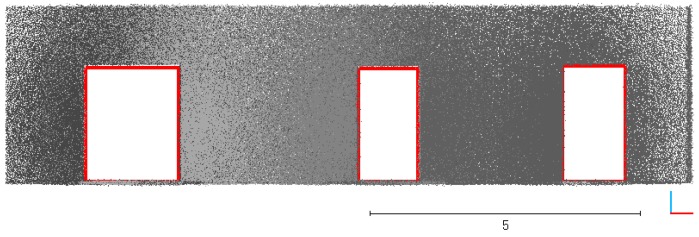
Hollow areas on the wall that were extracted as doors. Extracted areas are within red rectangles.

Four examples for U-V direction region growing are shown in Figure 9. The U-V growing algorithm first selects seed pixel s1-1 and then grows in the same row of the wall labeling image. Neighboring pixels that have similar properties are merged if the neighboring pixels’ value is empty. This growth step is repeated until all the pixels in the same row have been combined (i.e., growing in the U-direction, as the arrow shows). In the next row, the seed pixel s2-1 is then selected because it has the same value as s1-1 (i.e., seed selection in the V-direction). This growth step is repeated, similar to the process for the front row. In Figure 9, the red points show the start seeds of each row. The method of start-seed generation is seed selection from the front row. The hollow areas on the wall were extracted as doors as shown in Figure 10.

## 4. Experiments and Discussion

**Figure 11:**
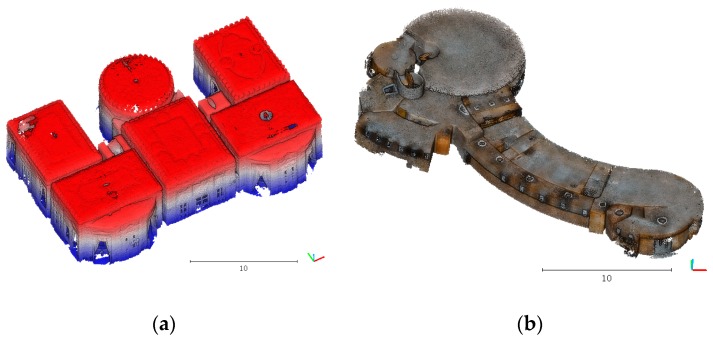
Test sites: original point clouds of a real-world dataset-1 (**a**) and dataset-2 (**b**).

The proposed method was tested on two real datasets of indoor scenes from the Matterport3D dataset [33]. The Matterport3D dataset is a large-scale RGB-D dataset containing 10,800 panoramic views from 194,400 RGB-D images of 90 building-scale scenes. The data were captured with a Matterport camera in home environments. The point clouds of these two datasets were created from raw data (Figure 11). The algorithm was implemented in C++. The algorithm was implemented by the Computational Geometry Algorithms Library (CGAL) [34] and Cloud Compare [35]. All experiments were performed with a 3.60 Hz Intel Core i7-4790 processor with 12 GB of RAM.

### 4.1. Wall Line Detection and Regularization

**Figure 12:**
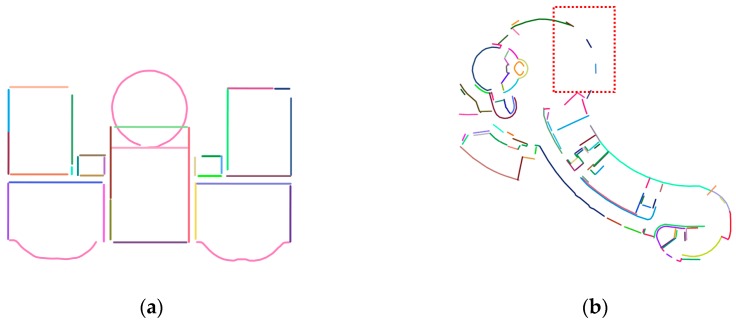
(**a**) Line tracing and regularization results for dataset-1; and (**b**) line tracing and regularization results for dataset-2. Because glass walls exist in dataset-2, the curved wall in the red box was detected as incomplete.

The point cloud is first split into several horizontal slices. The thickness of each slice is set as 0.01 m. The number of slices is influenced by the height and density of the input point cloud. The radius threshold $d_{1}=0.2\;m$ for searching the nearest neighbors was applied, and radius $d_{2}=0.1\;m$ was applied for marking points as visited. The wall line detection and regularization results for dataset-1 are shown in Figure 12a. The straight line regularization step was not used for dataset-2. As shown in Figure 12b, because glass walls exist in dataset-2, the curved wall was detected as incomplete.

### 4.2. Room Space Reconstruction

**Figure 13:**
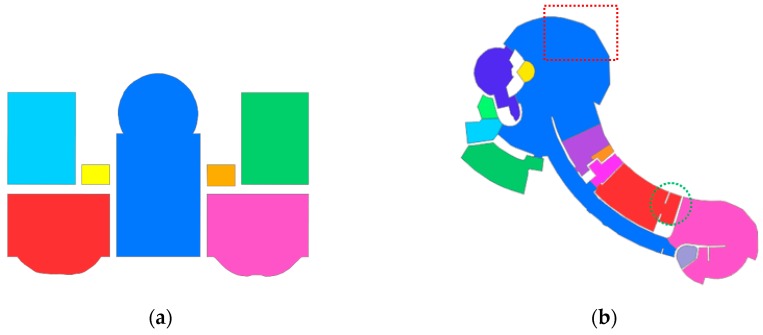
(**a**) Semantic decomposition of the room space and the generated floor plan for dataset-1; (**b**) semantic decomposition of the room space and the generated floor plan for dataset-2. Piecewise straight lines were used to approximate the curved line; the generated room boundary in red box was unsmooth.

The parameters of the room segmentation for different datasets are shown in Table 1. The same value of 25 mm was applied for the voxel size of the free space or pixel size of the 2D free-space evidence raster. The lower bound of the room area was the same. The upper bound of the room area was 125 m^2^ for dataset-1 and 75 m^2^ for dataset-2. Two-step cell complex construction was conducted. The 2D space was partitioned by straight line segments, and the temple result was further partitioned by curved lines. For dataset-2, the curved line segment was extended to ensure that the segment intersected with the existing polygon. A distance value of 0.5 m was used for this dataset. The room floor plan results of the two real-world datasets are shown in Figure 13. As shown in the red box in Figure 13b, because the curved wall was approximated by straight lines, the model result is not very smooth.

The geometric elements were evaluated quantitatively through a comparison between the reference model and the automatically reconstructed model, hereafter referred to as the source model. The intersection over union (IoU) metric, also known as the Jaccard similarity index, was used to evaluate the decomposition of room space. The IoU measures the similarity between finite sample sets and is defined as the ratio between the area of the intersection and union of the results and ground truth [6]. Similar to [3,5], the room discrepancy metric (RDM) was used to reflect the differences in the relative positions of wall corners and the Euclidean distances of all point pairs for the corners. The absolute area deviation (AAD) was also used in our evaluation to reflect the average room area differences.

(17) $$\mathrm{IoU}=\frac{\mathrm{Area}\;\mathrm{of}\;\mathrm{Intersection}}{\mathrm{Area}\;\mathrm{of}\;\mathrm{Union}}$$

(18) $$\mathrm{RDM}=\mathrm{dis}\left(P_{m}-{\;P}_{a}\right)$$

(19) $$\mathrm{AAD}=\left|{\mathrm{Area}}_{m}-{\;\mathrm{Area}}_{a}\right|$$

The experimental results are analyzed statistically in this section. As shown in Table 2, a total of 7 rooms were detected in dataset-1, and the IoU score of dataset-1 was 97.5% ± 2.4%. The proposed method obtained an RDM of 0.049 ± 0.031 m and an AAD of 0.35 ± 0.34 m^2^ for dataset-1. There were 13 rooms in the background. However, only 12 rooms were detected. Some unclear wall constraints from the point clouds caused the method to fail to determine the correct rooms. Due to the complexity of dataset-2, the accuracy was slightly low for this dataset. The IoU score of dataset-2 was 93.0% ± 6.85%. The proposed method obtained an RDM of 0.051 ± 0.025 m and an AAD of 0.49 ± 0.68 m^2^ for dataset-2.

### 4.3. Wall Opening Reconstruction

To estimate room height information, including the floor height, ceiling height, and room area, a histogram method was used to obtain the average height from the floor to the ceiling. In this experiment, the histogram interval was 0.05 m. The hollow areas on the wall were extracted. Four parameters were required in this step: the pixel size $s_{w}$, buffer threshold ε for the wall plane surface, upper bound of door widthr${\;w}_{0}$ and lower bound of door width $w_{1}$. First, the original points closer than the buffer threshold to the wall line were extracted. The buffer thresholds were set as 0.1 m in this study. Next, the points of each wall were projected to the wall planer surface to obtain a point evidence raster, and the U-V direction growing algorithm was used to extract hollow areas as openings. A value of 2.5 m was applied as the upper bound of the door width. A value of 0.3 m was applied as the lower bound of the door width.

**Figure 14:**
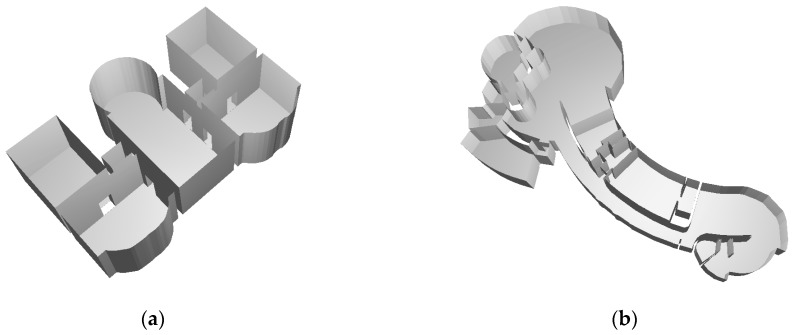
(**a**) Generated 3D indoor model for dataset-1; and (**b**) generated 3D indoor model for dataset-2. The ceilings are removed for clarity.

Finally, 3D models were completed, as shown in Figure 14. The total time consumptions were 74.9 and 123.8 s for dataset-1 and dataset-2, respectively (Table 3). As CityGML clearly defines the geometric and semantic relationships of builds, and the standard represents the building interior at LoD-4, the final 3D models were exported as the following file formats: *.obj file and CityGML LoD-4 (*.gml).

### 4.4. Discussion

**Figure 15:**
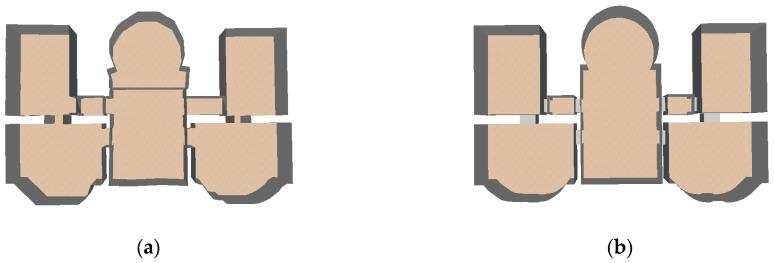
The comparison between the method using piecewise straight lines to approximate curved line (**a**) and proposed method (**b**).This study proposed a point-based method for curve detection of slice point cloud. The problem can also be solved in 2D domain by projecting the slice point cloud in XOY plane and using image-based method [36]. The image-based method often contains two steps, thinning [37,38] and tracing. It is useful and can detect curved lines quit well. However, the method still has two problems when compared with proposed method.

This study concentrated on the modeling of building interiors with curved walls. Similar to Li et al. [5], a few piecewise straight lines were used to approximate a curved line and an indoor 3D model was constructed. It can be clearly found that the generated room boundaries were not as smooth as proposed method (Figure 15).

**Figure 16:**
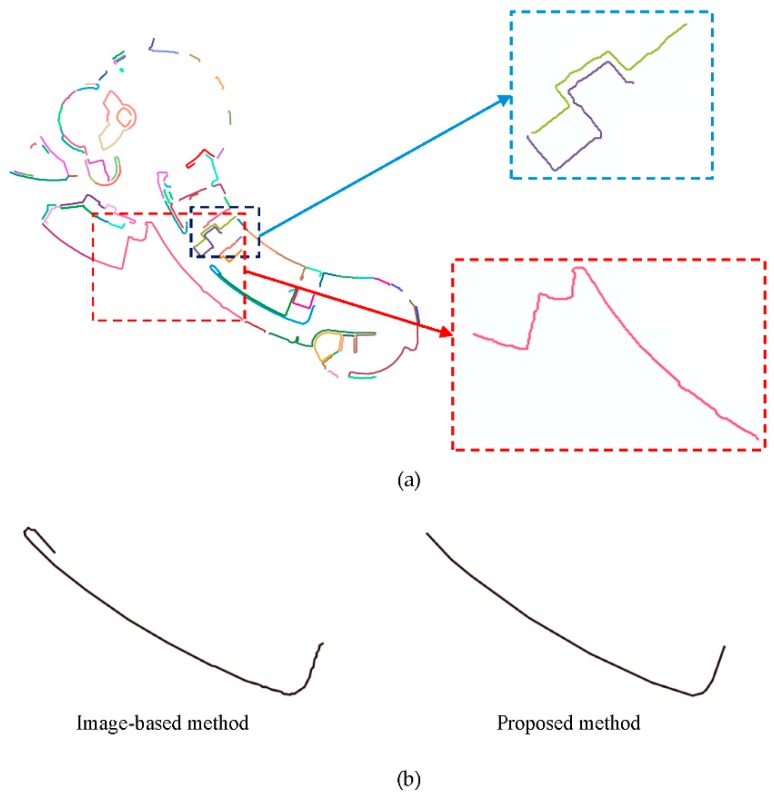
Curve detection result using image-based method. (**a**) The image-based method can’t distinct the straight parts and curved parts well; (**b**) the detected curves are not as smooth as proposed method.

(1) The image-based method can’t distinct the straight parts and curved parts well (Figure 16a).

(2) The detected curves are not as smooth as proposed method (Figure 16b).

The pixel size has big influence on curved line feature detection. For a larger cell size close to the thickness of the wall, the parallel wall lines won’t be detected. A smaller cell size will cause the thinning step to fail.

In the proposed method, vertical walls are assumed to exist in the multi-room indoor environment. However, some structural components, such as oblique walls, may oppose this assumption. Another difficulty arises from transparent glass walls and windows; dataset-2 contains many glass windows that influenced curved wall line detection, as shown in the red box of Figure 12b. While geometric elements are represented as volumetric solids in the IFC standard, the proposed method only models geometric elements as surfaces. Finally, only XYZ coordinates are used to detect wall opening elements in this study; therefore, more cues (e.g., color and intensity) can be used to detect wall opening elements and obtain more precise results.

## 5. Conclusions

In this work, a novel straight and curved line tracking method followed by a straight line test was proposed for curved wall detection. Robust parameters are used to represent the line direction parameter. A novel straight line regularization method is achieved using constrained least squares. By transforming indoor reconstruction into a labeling problem, the constructed cell complex with both straight lines and curved lines is labeled with graph cut based energy minimization. The method does not rely on viewpoint information. Instead, the clear wall constraints from point clouds were used in minus operation to determine the correct rooms in the initial room segmentation phrase. The experimental results indicated that the proposed method is well suited for indoor modeling of multi-room environments with curved walls.

In future work, the oblique wall issue must be solved. Only the geometric coordinates of points were used in this study; further work should include feature-based and vision combined methods for indoor reconstruction.

## Figures and Tables

**Table 1:** Parameters of the proposed method for different datasets.

Parameters	Descriptions	Dataset-1	Dataset-2
Wall line detection and regularization			
$d_{1}$	Search the nearest neighbor within a radius less than the threshold $d_{1}$.	0.2 m	0.2 m
$d_{2}$	The neighbor points within a radius $d_{2}$ are marked as visited.	0.1 m	0.1 m
k	The mean-shift runs k times, and each time the radius increases by 1.5 times.	3	3
Δθ	Straight and curved line detection angle threshold. (default 40°)	40°	40°
Δd	Straight line test distance threshold.	0.1 m	0.1 m
Δθ’	Straight line test angle threshold. (default 10°)	10°	10°
Room space reconstruction			
$s_{\mathrm{voxel}}{\;\mathrm{or}\;s}_{f}$	Voxel size of free space or pixel size of 2D free-space evidence raster, as well as point evidence raster.	25 mm	25 mm
$\tau_{\mathrm{lower}}$	The lower bound of a room area.	0.5 m^2^	0.5 m^2^
$\tau_{\mathrm{upper}}$	The upper bound of a room area.	125 m^2^	75 m^2^
Wall opening reconstruction			
$s_{w}$	Pixel size of wall labeling process, resolution of raster.	25 mm	25 mm
ε	Buffer threshold for the wall plane surface.	0.1 m	0.1 m
$w_{0}$	The upper bound of window/door width.	2.5 m	2.5 m
$w_{1}$	The lower bound of window/door width.	0.3 m	0.3 m

**Table 2:** Description of the room reconstruction evaluations using real-world datasets.

Test Sites		Background	Detected	IoU	RDM (m)	AAD (m^2^)
Dataset-1	Rooms	7	7	97.5% ± 2.4%	0.049 ± 0.031	0.35 ± 0.34
	Wall lines		36			
	Doors	8	8			
Dataset-2	Rooms	13	12	93.0% ± 6.85%	0.051 ± 0.025	0.49 ± 0.68
	Wall lines		91			
	Doors	7	7			

**Table 3:** The time consumption for each modeling phase.

Process Phase	Dataset-1	Dataset-2
Wall line detection and regularization (s)	6.1	15.9
Room space decomposition (s)	37.6	77.8
Wall opening detection (s)	31.2	30.1
Total time (s)	74.9	123.8

## References

[1] Wang C., Yong K.C., Kim C. (2015). Automatic BIM component extraction from point clouds of existing buildings for sustainability applications. Autom. Constr..

[2] (2012). OGC, OGC CityGML Encoding Standard, Document No.12-019. https://www.opengeospatial.org/standards/citygml.

[3] Macher H., Landes T., Grussenmeyer P. (2017). From Point Clouds to Building Information Models: 3D Semi-Automatic Reconstruction of Indoors of Existing Buildings. Appl. Sci..

[4] Previtali M., Díaz-Vilariño L., Scaioni M. (2018). Indoor Building Reconstruction from Occluded Point Clouds Using Graph-Cut and Ray-Tracing. Appl. Sci..

[5] Li L., Su F., Yang F., Zhu H., Li D., Zuo X., Li F., Liu Y., Ying S. (2018). Reconstruction of Three-Dimensional (3D) Indoor Interiors with Multiple Stories via Comprehensive Segmentation. Remote Sens..

[6] Ambruş R., Claici S., Wendt A. (2017). Automatic Room Segmentation From Unstructured 3D Data of Indoor Environments. IEEE Robot. Autom. Lett..

[7] Javidrad F., Pourmoayed A.R. (2011). Contour curve reconstruction from cloud data for rapid prototyping. Robot. Comput. Integr. Manuf..

[8] Lin Y., Cheng W., Cheng J., Chen B., Jia F., Chen Z., Li J. (2015). Line segment extraction for large scale unorganized point clouds. ISPRS J. Photogramm. Remote Sens..

[9] Sanchez V., Zakhor A. Planar 3D modeling of building interiors from point cloud data. Proceedings of the IEEE International Conference on Image Processing.

[10] Adan A., Huber D. 3D Reconstruction of Interior Wall Surfaces under Occlusion and Clutter. Proceedings of the International Conference on 3D Imaging, Modeling, Processing, Visualization and Transmission.

[11] Shi W., Ahmed W., Li N., Fan W., Xiang H., Wang M. (2019). Semantic Geometric Modelling of Unstructured Indoor Point Cloud. ISPRS Int. J. Geo-Inf..

[12] Ikehata S., Yang H., Furukawa Y. Structured Indoor Modeling. Proceedings of the IEEE International Conference on Computer Vision.

[13] Oesau S., Lafarge F., Alliez P. (2014). Indoor scene reconstruction using feature sensitive primitive extraction and graph-cut. ISPRS J. Photogramm. Remote Sens..

[14] Mura C., Mattauscha O., Villanuevab J.A., Gobbetti E., Pajarolaa R. (2014). Automatic room detection and reconstruction in cluttered indoor environments with complex room layouts. Comput. Graph..

[15] Xie L., Wang R., Chen D., Xie L., Wang R., Chen D. (2017). Modeling Indoor Spaces via Decomposition and Reconstruction of Structural Elements. Photogramm. Eng. Remote Sens..

[16] Sareen K.K., Knopf G.K., Canas R. Surface reconstruction from sliced point cloud data for designing facial prosthesis. Proceedings of the IEEE Toronto International Conference Science and Technology for Humanity (TIC-STH).

[17] Robbins B.J. (1996). The Detection of 2D Image Features Using Local Energy. Ph.D. Thesis.

[18] Mcilhagga W. (2011). The Canny Edge Detector Revisited. Int. J. Comput. Vis..

[19] Schnabel R., Wahl R., Klein R. (2007). Efficient RANSAC for Point-Cloud Shape Detection. Comput. Graph. Forum.

[20] Fischler M.A., Bolles R.C. (1981). Random sample consensus: A paradigm for model fitting with applications to image analysis and automated cartography. Commun. ACM.

[21] Borrmann D., Elseberg J., Kai L., Nüchter A. (2011). The 3D Hough Transform for plane detection in point clouds: A review and a new accumulator design. 3D Res..

[22] Peter M., Jafri S.R.U.N., Vosselman G. (2017). Line segmentation of 2d laser scanner point clouds for indoor slam based on a range of residuals. ISPRS Ann. Photogramm. Remote Sens. Spat. Inf. Sci..

[23] Yang B., Zang Y. (2014). Automated registration of dense terrestrial laser-scanning point clouds using curves. ISPRS J. Photogramm. Remote Sens..

[24] Wang W., Pottmann H., Yang L. (2006). Fitting B-Spline Curves to Point Clouds by Squared Distance Minimization. ACM Trans. Graph..

[25] Rusu R.B. (2010). Semantic 3D Object Maps for Everyday Manipulation in Human Living Environments. KI-Künstl. Intell..

[26] Verdie Y., Lafarge F., Alliez P. (2015). LOD Generation for Urban Scenes. ACM Trans. Graph..

[27] Jung J., Stachniss C., Kim C. (2017). Automatic Room Segmentation of 3D Laser Data Using Morphological Processing. Int. J. Geo-Inf..

[28] Li L., Li D., Xing X., Yang F., Rong W., Zhu H. (2017). Extraction of Road Intersections from GPS Traces Based on the Dominant Orientations of Roads. ISPRS Int. J. Geo-Inf..

[29] Li G., Huang H., Wu S., Cohen-Or D., Chen B. (2013). L1-medial skeleton of point cloud. ACM Trans. Graph..

[30] Bormann R., Jordan F., Li W., Hampp J., Hýgele M. Room segmentation: Survey, implementation, and analysis. Proceedings of the IEEE International Conference on Robotics and Automation.

[31] Ritter G.X., Wilson J.N. (2001). Handbook of Computer Vision Algorithms in Image Algebra.

[32] Boykov Y., Veksler O., Zabih R. Fast approximate energy minimization via graph cuts. Proceedings of the IEEE Transactions on Pattern Analysis and Machine Intelligence.

[33] Chang A., Dai A., Funkhouser T., Halber M., Nießner M., Savva M., Song S., Zeng A., Zhang Y. Matterport3D: Learning from RGB-D Data in Indoor Environments. Proceedings of the International Conference on 3D Vision (3DV).

[34] CGAL. https://www.cgal.org/.

[35] CloudCompare. http://www.cloudcompare.org/.

[36] ESRI ArcGIS. https://www.arcgis.com/index.html.

[37] Zhang T.Y., Suen C.Y. (1984). A Fast Parallel Algorithm for Thinning Digital Patterns. Commun. ACM.

[38] Lam L., Lee S.W., Suen C.Y. (1992). Thinning Methodologies-A Comprehensive Survey. IEEE Trans. Pattern Anal. Mach. Intell..

